# NCAPG Promotes the Proliferation of Renal Clear Cell Carcinoma via Mediating with CDK1

**DOI:** 10.1155/2022/6758595

**Published:** 2022-05-13

**Authors:** Huibing Li, Pengyi Zheng, Zhijun Li, Qingjiang Han, Bisheng Zhou, Xiaohui Wang, Kaixuan Wang

**Affiliations:** Department of Urologic Surgery, The First Affiliated Hospital and College of Clinical Medicine of Henan University of Science and Technology, Luoyang, 471003 Henan, China

## Abstract

**Objective:**

Currently, lots of scholars have proved that the expression of NCAPG is associated with the prognosis of several cancers, while the relationship between NCAPG and renal clear cell carcinoma remains unclear, so the main aim of this research is to explore the effects of NCAPG on the progression of renal clear cell carcinoma.

**Methods:**

We observed the differential expression of NCAPG in several cancers from GEPIA online database, and the expression of NCAPG in renal clear cell carcinoma and normal tissue was compared and further verified by IHC assay. CCK-8 assay and clone formation experiment were conducted to observe the change of NCAPG on the proliferation. GraphPad was used for data analysis, and *t*-test and *χ*^2^ analysis were used to analyze the correlation between NCAPG/CDK1 and renal clear cell carcinoma.

**Results:**

NCAPG was upregulated in renal clear cell carcinoma compared with the normal tissue, and the expression of NCAPG was associated with the clinical prognosis of pancreatic cancer especially with tumor size (*P* = 0.010). Knockdown NCAPG could restrain the proliferation of renal clear cell carcinoma. CDK1 was found to be tightly related with NCAPG, and the expression of CDK1 was also associated with the prognosis.

**Conclusions:**

NCAPG was upregulated in renal clear cell carcinoma, which was related with tumor size and overall survival. NCAPG might promote the proliferation of renal clear cell carcinoma via mediating CDK1. NCAPG/CDK1 complex might provide a new treatment strategy for lots of patients with renal clear cell carcinoma.

## 1. Background

Currently, renal clear cell carcinoma has been known as one of the most common malignant cancers all over the world, and the increasing incidence of renal clear cell carcinoma is becoming critical and tricky issue for lots of patients with renal clear cell carcinoma [1]. According to the cancer statistics in 2020 [2], there were approximately 73750 new cases with renal clear cell carcinoma, and what interested us was that the percentage of male patients was 1.5 times of female patients. The results further showed that the morbidity rate of renal clear cell carcinoma in male ranked sixth among all kinds of cancers and ranked eighth in female. Although the mortality rate of renal clear cell carcinoma ranked behind tenth among all cancers, there were still 14830 death cases owing to renal clear cell carcinoma [3]. With the development of medicine and surgery, the cure rate and survival rate have obtained huge improvement, and surgical resection was still the nonsubstitutable and most important treatment measure [4], coupled with radiation therapy, chemotherapy, targeted therapy, and immunity therapy that make lots of patients obtain a longer survival rate [5, 6]. However, the diagnosis of renal clear cell carcinoma still lacked a sensitive biomarker, and it was becoming extremely urgent for us to find a new treatment strategy for many patients with renal clear cell carcinoma.

NCAPG (non SMC condensin I complex subunit G) is a subunit of the lectin complex which is responsible for the concentration and stability of chromosomes during mitosis and meiosis and protein phosphorylation activation polysaccharide composition [7, 8]. The dysregulation of NCAPG was associated with the occurrence of many diseases, including endometrial mixed adenocarcinoma and croup. Previous researches have found that NCAPG was tightly related with the occurrence and development of several cancers, such as Gong et al. who found that NCAPG promoted the proliferation of hepatocellular carcinoma through PI3K/AKT signaling [9] and Zhang et al. also who identified that NCAPG could induce the proliferation in cardia adenocarcinoma via PI3K/AKT signaling pathway [10]. Xiao et al. identified that NCAPG was a promising therapeutic target across different tumor types [11], which implied that NCAPG was a critical factor for the treatment of various cancers. Moreover, Sun et al. found that aberrant expression of NCAPG is associated with prognosis and progression of gastric cancer [12]. The role of NCAPG in the progression and proliferation of various cancers has been confirmed. However, the relationship between NCAPG and renal clear cell carcinoma remains unclear. It has been known that NCAPG was associated with mitosis and meiosis and whether NCAPG was related with cell cycle related factors. CDK1 (cyclin-dependent kinase 1) was a member of the Ser/Thr protein kinase family and was essential for G1/S and G2/M phase transitions of eukaryotic cell cycle [13]. Previous researches of CDK1 have found that the dysregulation of CDK1 was tightly related with the occurrence and evolution of many diseases. Many researchers have found that CDK1 was tightly associated with the occurrence and development of several cancers. For example, Xie et al. found that cyclin B1/CDK1-regulated mitochondrial bioenergetics in cell cycle progression and tumor resistance [14], and Ravindran Menon et al. also found that CDK1 interacts with Sox2 and promotes tumor initiation in human melanoma [15]. Moreover, Li et al. identified that CDK1 served as a potential prognostic biomarker and target for lung cancer [16]. As a result, the role of CDK1 in renal clear cell carcinoma still remained unclear, and the relationship between NCAPG and CDK1 in renal clear cell carcinoma needs further exploration. The main purpose of this study is to preliminarily explore the correlation between renal clear cell carcinoma and NCAPG and CDK1.

## 2. Methods

### 2.1. The Information of Patients

All patients with renal clear cell carcinoma involved in this research have provided informed consent. These tissues of the 72 cases are all derived from our hospital, and the sampling process was performed by scientific methods.

### 2.2. Cells and Transfection

Normal kidney cell HK-2 was purchased from ATCC, which were all cultured with K-SFM complete medium; renal clear cell carcinoma HTB-47 and CRL-1932 cells were purchased from ATCC, which were all cultured with 1640 containing 10% FBS and 1% penicillin-streptomycin double antibody in the incubator with 37°C and 5%CO_2_. We transfected HTB-47 and CRL-1932 cells with designed NCAPG, CDK1 shRNA, and CDK1 overexpression plasmid according to transfection reagent. These designed NCAPG shRNA sequences were presented: shRNA 1: CCAGAACCAGGCGAAGCTGGTGG; shRNA 2: AAAGACTTTGCCAAAAATTGTAG; shRNA 3: AAAAGAATTCATAGGTCAACAAT; and shRNA 4: TTTGATTCTTCCTGGAATAATAA. And CDK1 shRNA sequences were presented: shRNA 1: CAGGTTATATCTCATCTTT; shRNA 2: ′-GCTTGGATTTGCTCTCGAA and shRNA 3: GGAATCTTTACAGGACTAT. A total of 6 × 10^5^ HTB-47 and CRL-1932 cells were seeded in 6-well plates. After 24 h, the cells were transfected with 2 *μ*g plasmid or 100 nM siRNA with Lipofectamine™ 2000 (Invitrogen) according to the manufacturer's protocol. The related plasmid was transfected into HTB-47 and CRL-1932 cells. The knockdown of NCAPG and CDK1 was generated by transfection with related shRNA.

### 2.3. Reagents and Antibodies

Western blotting antibodies are as follows: anti-NCAPG (ab251864) rabbit 1 : 1000; anti-CDK1 (ab201008), rabbit, 1 : 1000; and anti-GAPDH (ab8245) mouse 1 : 1000. immunohistochemical staining antibodies are as follows: anti-NCAPG (ab251864) rabbit 1 : 100 and anti-CDK1 (ab201008), rabbit, 1 : 100.

### 2.4. Extracting Database

We followed previous retrieval strategy [17] and extracted the differential expression and overall survival of NCAPG in renal clear cell carcinoma and normal tissue from GEPIA online database (http://gepia.cancer-pku.cn/detail.php?gene=NCAPG). The expression of NCAPG in several cancers was analyzed in the online database (https://www.proteinatlas.org/ENSG00000109805-NCAPG/tissue). The expression and prognosis of CDK1 in renal clear cell carcinoma were analyzed from GEPIA online database (http://gepia.cancer-pku.cn/detail.php?gene=CDK1). The expression level of CDK1 in several cancers was analyzed from online database (https://www.proteinatlas.org/ENSG00000170312-CDK1/tissue).

### 2.5. RNA Extracting and PCR

The total RNA was extracted from HTB-47 and CRL-1932 cells transfected with NCAPG shRNA, which was used to observe the mRNA expression of NCAPG in NCAPG knockdown group and the control group.

### 2.6. Western Blotting

The total protein was extracted from HTB-47 and CRL-1932 cells transfected with NCAPG shRNA and CDK1 shRNA, which was used to observe the protein expression of NCAPG, CDK1, and GAPDH based on the western blotting assay. Total protein was extracted from HTB-47, CRL-1932 cell lines, and tumor tissues using RIPA (Biosharp) and PMSF, and the BCA kit was used to determine the protein concentration. In the 10% acrylamide gels, an equal amount of protein sample (40 *μ*g per channel) was separated by SDS-polyacrylamide gel electrophoresis (PAGE) and transferred to the polyvinylidinedifluoride (PVDF) membrane. The membrane was blocked in 5% fat-free milk and incubated overnight with the above-mentioned antibodies. Then, the PVDF membrane was washed and incubated with IgG for 1 h at room temperature. The immunoreactive bands were detected by chemiluminescence methods and visualized using luminescent imaging workstation.

### 2.7. Immunohistochemical Assay

Immunohistochemical assay was performed based on the immunohistochemical staining kit, and these paraffin specimens were sliced at 3-4 *μ*m and grilled slices at 70°C for 45 minutes and then soaked in the following order: xylene solution (1) for 10 min, xylene solution (2) for 10 min, absolute ethanol solution (1) for 5 min, absolute ethanol solution (2) for 5 min, 95% alcohol solution for 5 min, 85% alcohol solution for 5 min, and 75% alcohol solution for 5 min. We washed them in PBS buffer for 2 minutes two times and then placed the slices in sodium citrate buffer in the microwave. Next, we placed them at room temperature for 2 hours and washed with PBS buffer for 2 min two times. Then, we removed excess water and dropped H_2_O_2_ in the wet box for 20 min at room temperature, and after that, we washed them again and dropped BUB3 and CDCA3 antibodies for 4°C. In the next day, we placed them at room temperature for one hour and washed them with PBS buffer for 2 min two times, and then, we dropped IgG secondary antibodies for 2 hours at room temperature and washed them again with PBS buffer. At last, we dropped DAB (preformulated with liquid A and liquid B) and stained at room temperature about 5-15 seconds. Stop dyeing with water, and next, stain nucleus with hematoxylin solution for 5-8 seconds and tap water rinse for 2 min. We should soak in following order: 75% alcohol solution, 85% alcohol solution, and 95% alcohol solution each for 1 min, absolute ethanol (1) and absolute ethanol (2) each for 3 min, and xylene (1) and xylene (2) each for 5 min. Finally, neutral gum sealer and look under the microscope.

### 2.8. Colony Forming Assays

We planted 2.5∗10^3^ cells of HTB-47 and CRL-1932 transfected with NCAPG shRNA into 6-well plate, and these cells were cultured for additional week. Finally, we fixed these cells with 4% paraformaldehyde and stain with crystal violet.

### 2.9. CCK-8 Assay

The 3∗10^3^ cells of HTB-47 and CRL-1932 were transfected with NCAPG shRNA into a 96-well plate, which were cultured for additional 3 days. We finally added CCK-8 reagent and observed 570 nm absorbance.

### 2.10. Statistical Analysis

We used software GraphPad Prism 7 to analyze data and results. *t*-test and *χ*^2^ analysis were conducted to analyze the correlation between NCAPG and CDK1 and the comparison between the two groups.

## 3. Results

### 3.1. High Expression of NCAPG in Several Cancers

**Figure 1:**
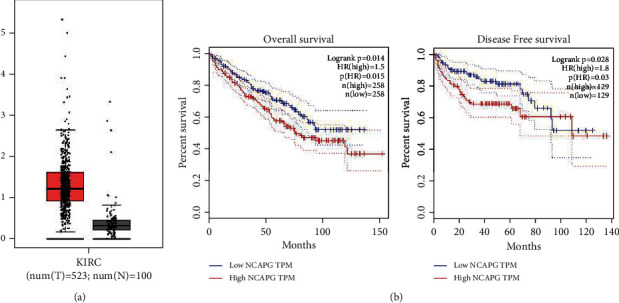
The expression level of NCAPG is positively correlated with the poor prognosis of patients with renal clear cell carcinoma. (a) The expression of NCAPG in renal clear cell carcinoma and normal tissues in GEPIA database; the expression of cancer is obviously higher than in the normal tissues. (b) Overall and disease-free survival rates of low and high expression NCAPG; higher the expression of NCAPG, lower overall and disease-free survival rates in renal clear cell carcinoma patients.

Previous studies have proved that the expression of NCAPG was tightly associated with the occurrence and development of several cancers. To further verify the results, we extracted the differential expression of NCAPG in several common cancers from online database, including colorectal cancer, breast cancer, and lung cancer. The results showed that the expression of NCAPG was upregulated in colorectal cancer, breast cancer, and prostate cancer while weak expression in lung cancer (Figure 1(a)). And we further found that NCAPG displayed moderate cytoplasmic positivity, especially strongly staining in lymphoma, testicular, colorectal, and endometrial cancers while weakly stained in gliomas and prostate cancer (Figure 1(b)). The results were consistent with the conclusion of the previous researches.

### 3.2. NCAPG Is Upregulated in Renal Clear Cell Carcinoma and Related with Prognosis

**Figure 2:**
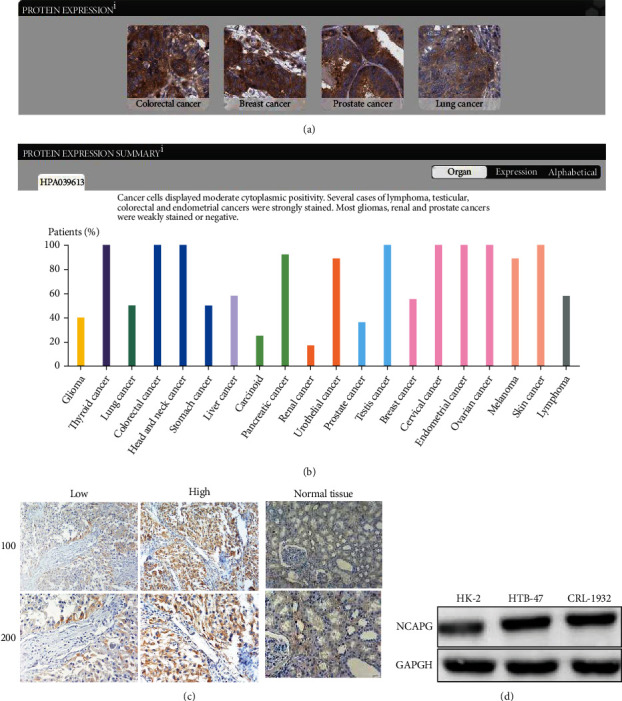
High expression of NCAPG in renal clear cell carcinoma. (a) The expression of NCAPG in colorectal cancer, breast cancer, prostate cancer, and lung cancer by IHC staining. (b) The expression of NCAPG displayed moderate nuclear and cytoplasmic staining in various cancers. (c) The expression of NCAPG is upregulated in renal clear cell carcinoma compared with normal tissue. (d) The protein expression of NCAPG in normal kidney cell HK-2 and renal cell carcinoma cells HTB-47 and CRL-1932.

To further explore the expression of NCAPG in renal clear cell carcinoma and the normal tissue, we extracted the differential expression of NCAPG in renal clear cell carcinoma and the normal tissue from GEPIA online database. The results showed that the expression of NCAPG was significantly higher in renal clear cell carcinoma compared with the normal tissue (Figure 2(a)). We further found that the expression of NCAPG was associated with the overall survival and disease-free survival of renal clear cell carcinoma, and these patients with higher expression of NCAPG have a poorer OS and DFS (Figure 2(b)). To further verify the conclusion from the database, we collected 72 paired tissue of patients with renal clear cell carcinoma and conducted immunohistochemical staining to observe the expression of NCAPG. The results of IHC assay were consistent with the results of database (Figure 2(c)), and we further divided 72 patients into high and low groups based on the staining intensity of NCAPG. Next, we explore the correlation between the expression of NCAPG and several common clinicopathological parameters of renal clear cell carcinoma, including age, gender, tumor grade, and tumor size. The results showed that the expression of NCAPG was tightly related with tumor size (*P* = 0.010) while not related with age, gender, and tumor grade (*P* > 0.05; Table 1). Finally, we further explore NCAPG expression in normal kidney cell HK-2 and renal cell carcinoma cell HTB-47 and CRL-1932 cells, and the results showed that NCAPG expression was significantly elevated in renal cell carcinoma cells compared with the normal kidney cell (Figure 2(d)). All the above results implied that the expression of NCAPG was upregulated in renal clear cell carcinoma and related with tumor size. NCAPG might provide a new diagnosis and prognosis-related biomarker for lots of patients with renal clear cell carcinoma.

### 3.3. Knockdown NCAPG Restrained the Proliferation of Renal Clear Cell Carcinoma

**Figure 3:**
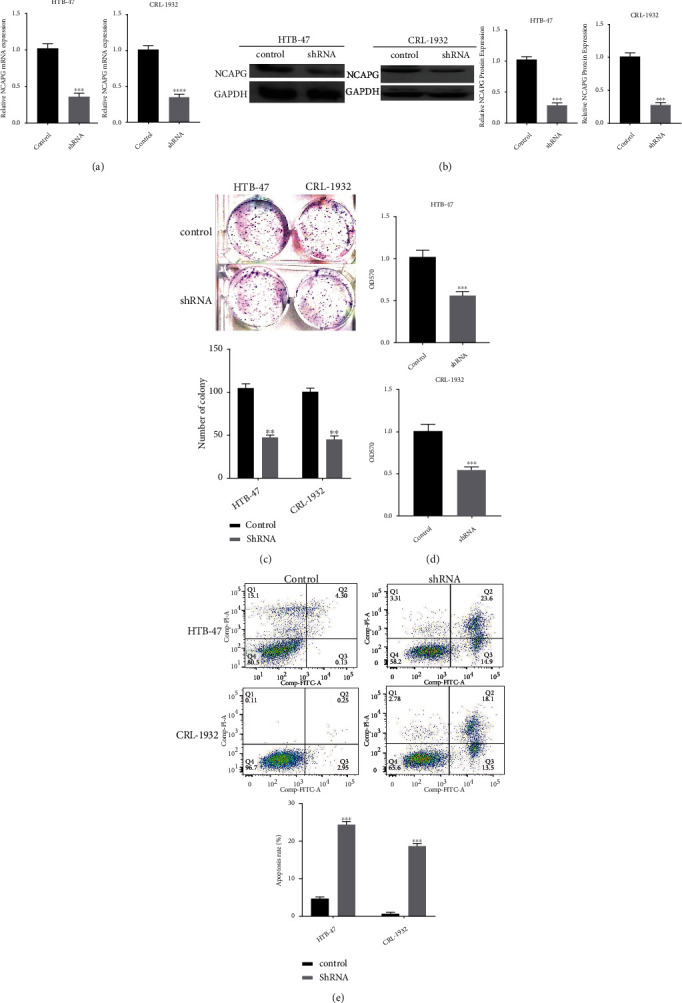
Knockdown NCAPG could restrain the proliferation of renal clear cell carcinoma HTB-47 and CRL-1932 cells. (a) The mRNA expression of NCAPG in HTB-47 and CRL-1932 cells transfected with NCAPG shRNA. (b) The protein expression of NCAPG in HTB-47 and CRL-1932 cells transfected with NCAPG shRNA. (c) The colony forming assay of HTB-47 and CRL-1932 cells transfected with NCAPG shRNA. (d) The CCK-8 assay of HTB-47 and CRL-1932 cells transfected with NCAPG shRNA. (e) The apoptosis assay of HTB-47 and CRL-1932 cells transfected with NCAPG shRNA.

To further consolidate the hypothesis that NCAPG promoted the progression of renal clear cell carcinoma, we explored the effects of NCAPG on the proliferation of renal clear cell carcinoma. We transfected renal clear cell carcinoma HTB-47 and CRL-1932 cells with designed four NCAPG shRNA, and then, we selected the best one for subsequent experiments. Firstly, we extracted total RNA from HTB-47 and CRL-1932 cells transfected with NCAPG shRNA, and the results displayed that the mRNA expression of NCAPG was restrained obviously in NCAPG knockdown group compared with the normal group (Figure 3(a)). We continued to explore the protein change of NCAPG and found that the protein level of NCAPG declined significantly in NCAPG shRNA group than control group (Figure 3(b)). We further explored the effects of knockdown NCAPG on the proliferation and progression of renal clear cell carcinoma; we performed CCK-8 assay and colony forming assay to observe the change of proliferation ability in HTB-47 and CRL-1932 cells. And the results of colony forming assay demonstrated that the colony forming ability of HTB-47 and CRL-1932 cells was restrained significantly in NCAPG shRNA group compared with control group (Figure 3(c)). And the results of CCK-8 assay further consolidated the conclusion; we found that knockdown NCAPG could restrain significantly the proliferation ability compared with the control group (Figure 3(d)). We further found that NCAPG shRNA also promotes the apoptosis rate of HTB-47 and CRL-1932 cells compared with the control group (Figure 3(e)), which further implied that NCAPG regulated the progression of renal clear cell cancer which depend not only proliferation but also apoptosis. All the above results showed that NCAPG promoted the proliferation and progression of renal clear cell carcinoma and related with the prognosis. NCAPG might provide a new diagnosis-related biomarker and therapeutic target for lots of patients with renal clear cell carcinoma.

### 3.4. CDK1 Was Associated with NCAPG and Prognosis of Renal Clear Cell Carcinoma

**Figure 4:**
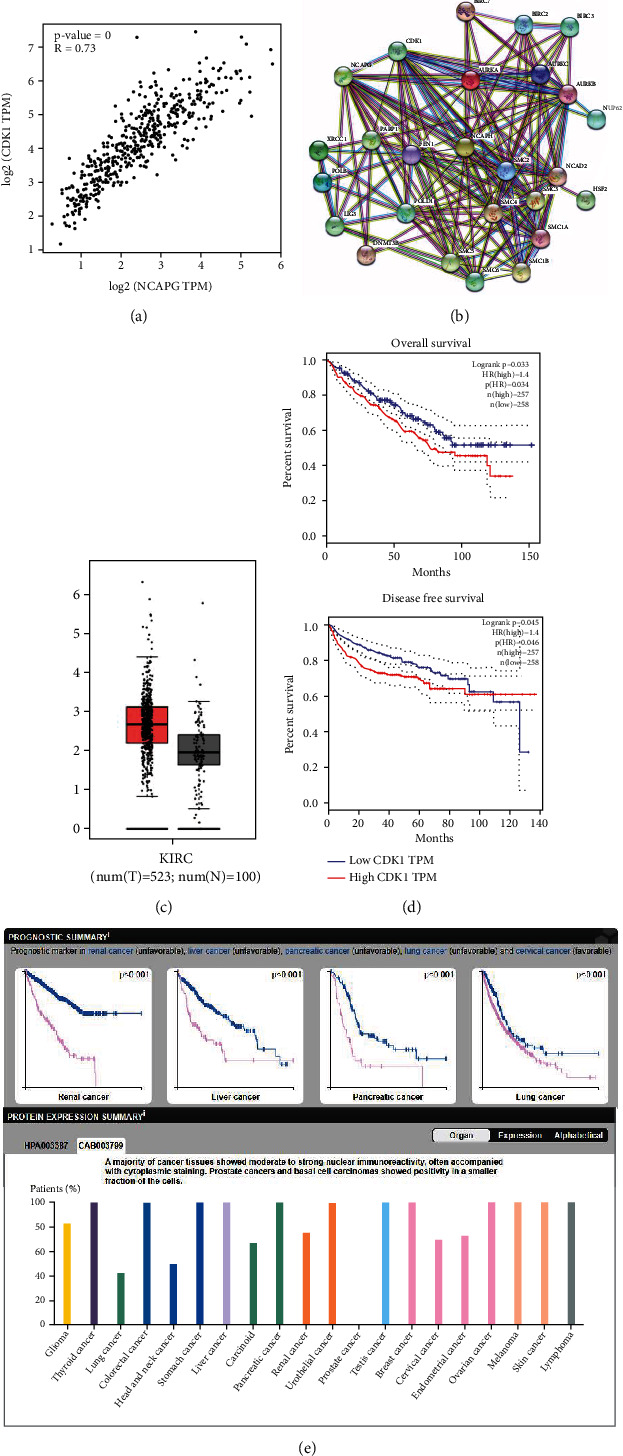
CDK1 is upregulated in renal clear cell carcinoma and related with the prognosis. (a) The correlation between NCAPG and CDK1 via online database. (b) The expression of CDK1 in renal clear cell carcinoma and normal tissues in GEPIA database; the expression of cancer is obviously higher in renal clear cell carcinoma. (c) Overall survival rates and disease-free survival rate of low and high expression CDK1 in renal clear cell carcinoma. (d) The network analysis of CDK1 and NCAPG. (e) The expression of CDK1 is upregulated in renal clear cell carcinoma compared with normal tissue.

It has been known that CDK1 was upregulated in several cancers and associated with the prognosis and progression. To further explore the correlation between CDK1 and NCAPG in renal clear cell carcinoma, we extracted the correlation between NCAPG and CDK1 from online database, and the results showed that CDK1 was positively related with NCAPG (*R* = 0.73; *P* < 0.01; Figure 4(a)). To further explore the functional possibility of NCAPG interacting with CDK1, we predicted the correlation between CDK1 and other factors based on the online database; the results demonstrated that CDK1 interacted with NCAPG (Figure 4(b)). We further found that the expression of CDK1 was upregulated in renal clear cell carcinoma compared with normal tissue (Figure 4(c)), and the expression of NCAPG was associated tightly with overall survival and disease-free survival in renal clear cell carcinoma (Figure 4(d)), which was consistent with the results of NCAPG. Previous studies proved that CDK1 was a diagnosis biomarker for several cancers; we predicted the diagnostic value of CDK1 in renal cancer, liver cancer, pancreatic cancer, and lung cancer from online database; the results showed that CDK1 was a good prognostic biomarker (*P* < 0.01; Figure 4(e)). The results of the database demonstrated that CDK1 was moderate to strong nuclear staining in majority cancers (Figure 4(f)). All the above results showed that CDK1 was upregulated in renal clear cell carcinoma and associated with prognosis and NCAPG; NCAPG might promote the proliferation and progression of renal clear cell carcinoma via mediating CDK1.

### 3.5. NCAPG Promotes the Progression of Renal Clear Cell Carcinoma via Regulating CDK1

**Figure 5:**
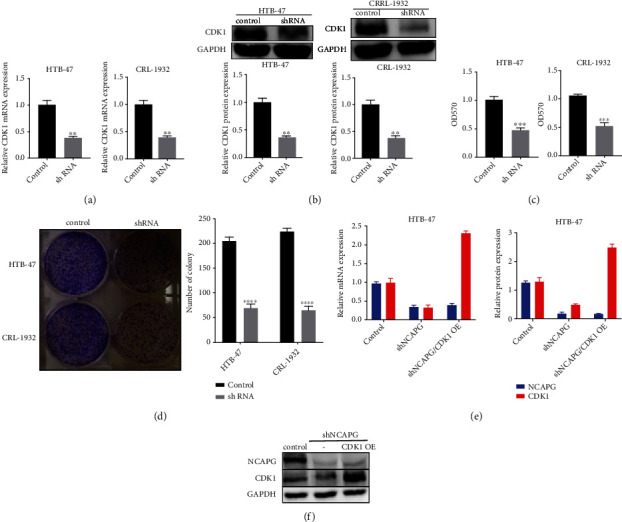
NCAPG promotes the progression of renal clear cell carcinoma via regulating CDK1. (a) The mRNA expression of CDK1 in HTB-47 and CRL-1932 cells transfected with CDK1 shRNA. (b) The protein expression of CDK1 in HTB-47 and CRL-1932 cells transfected with CDK1 shRNA. (c) The CCK-8 assay of HTB-47 and CRL-1932 cells transfected with CDK1 shRNA. (d) The colony forming assay of HTB-47 and CRL-1932 cells transfected with CDK1 shRNA. (e) The mRNA expression of CDK1 and NCAPG in HTB-47 transfected with NCAPG shRNA and CDK1 overexpression plasmid. (f) The protein expression of CDK1 and NCAPG in HTB-47 transfected with NCAPG shRNA and CDK1 overexpression plasmid.

**Figure 6:**
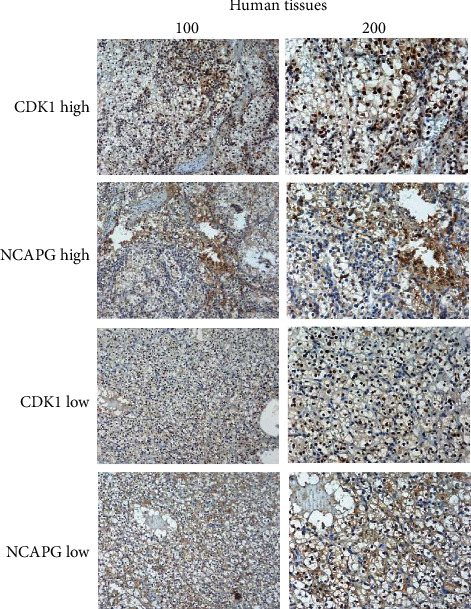
The correlation between NCAPG and CDK1 via immunohistochemical staining. These cases with high expression of NCAPG have similar high expression level of CDK1.

To further explore the relationship between NCAPG and CDK1, we explored the effects of CDK1 on the proliferation of renal clear cell carcinoma. Firstly, we transfected HTB-47 and CRL-1932 cells with designed CDK1 shRNA and extracted total RNA from HTB-47 and CRL-1932 cells; the results displayed that the mRNA expression of CDK1 was restrained obviously in CDK1 knockdown group compared with the control group (Figure 5(a)). We continued exploring the protein change of CDK1 and found that the protein level of CDK1 was restrained in CDK1 shRNA group compared with control group (Figure 5(b)). Further, we explored the effects of knockdown CDK1 on the proliferation and progression of renal clear cell carcinoma; CCK-8 assay and colony forming assay were performed to observe the change of proliferation ability in HTB-47 and CRL-1932 cells. And the results of colony forming assay and CCK-8 assay demonstrated that the proliferation ability of HTB-47 and CRL-1932 cells was restrained significantly in CDK1 shRNA group compared with control group (Figures 5(c) and 5(d)). These results further implied that CDK1 has the similar effects on the progression of renal clear cell carcinoma with NCAPG. To further explore the regulatory network between NCAPG and CDK1, we explored a rescue experiment to verify the hypothesis. The results showed that CDK1 expression could be restrained by NCAPG shRNA and recovered by CDK1 overexpression, but NCAPG expression could not be recovered liking CDK1 (Figures 5(e) and 5(f)). The results consolidated the hypothesis that NCAPG promotes the proliferation and progression of renal clear cell carcinoma via mediating CDK1. To further consolidate all the above results and the hypothesis that NCAPG promoted the proliferation and progression via mediating with CDK1, we tried to analyze the relationship between the expression of NCAPG and the expression of CDK1 among 72 cases with renal clear cell carcinoma; we found that CDK1 was associated with NCAPG (*P* = 0.001; Table 2). And these results were consistent with the results of the database; we further analyzed the correlation between NCAPG and CDK1 by immunohistochemical staining; the results showed that these patients with high expression of NCAPG almost have a similar high expression of CDK1 (Figure 6). Finally, we have reasons to believe that NCAPG promoted the proliferation and progression of renal clear cell carcinoma via mediating CDK1, and NCAPG/CDK1 complex might provide a new strategy for the treatment of renal clear cell carcinoma.

## 4. Discussion

Due to the high incidence rate and rapid development of renal clear cell carcinoma, lots of patients lost the choices of completely cured or surgical resection, part of whom were diagnosed at late stage as a result of no obvious symptoms [4]. Although surgical resection increased significantly the survival rate and other adjuvant therapy further improved the prognosis of lots of patients, it was still urgent to look for a diagnosis-related biomarker [18]. Currently, these existing diagnosis-related measures or biomarkers lacked specificity and sensitivity, for example, the value of CEA or ultrasonography [19, 20]. An early diagnosis-related biomarker was more and more urgent for lots of patients with renal clear cell carcinoma. Many scholars have researched and proved that NCAPG is a mitotic-associated aggregation protein, consisting of approximately 1015 amino acids and a molecular weight of approximately 114.1 KDa [21, 22]. Previous researches have found that NCAPG was related tightly with the occurrence and progression of serious cancers and might provide a sensitive diagnosis-related biomarker for several cancers. In this study, we found that NCAPG was upregulated in several cancers, including in renal clear cell carcinoma. We further found that the expression of NCAPG was associated tightly with the prognosis and progression of renal clear cell carcinoma. We still found that the expression of NCAPG was associated with the tumor size of renal clear cell carcinoma by analyzing the relationship between NCAPG and several clinicopathological parameters among 72 patients with renal clear cell carcinoma. It was interested that Xu et al. identified that the elevated mRNA expression levels of NCAPG were associated with poor prognosis in ovarian cancer [23]. And Zhang et al. also found that overexpression of NCAPG could inhibit the apoptosis of cardia adenocarcinoma and promote epithelial-mesenchymal transition (EMT) through the Wnt/*β*-catenin signaling pathway [24]. To further explore the effects of NCAPG in the proliferation and progression of renal clear cell carcinoma, we knockdown NCAPG and found that NCAPG could promote significantly the proliferation and progression of renal clear cell carcinoma. It was consistent with the results of Wang et al., who identified that genome-wide CRISPR knockout screens identify NCAPG as an essential oncogene for hepatocellular carcinoma tumor growth [25]. And Zhang et al. also found that NCAPG was a novel mitotic gene required for hepatocellular cancer cell proliferation and migration [26]. There were so many articles indicated that NCAPG was upregulated in various cancers and promoted the proliferation and progression, which further consolidate our results of NCAPG promoting the proliferation of renal clear cell carcinoma. The effects of NCAPG on the invasion ability of renal clear cell carcinoma remain unclear, which will be the next aim of our research. To further explore the mechanism of NCAPG promoting the proliferation and progression, we identified that NCAPG was correlated tightly with CDK1 based on database; we further found that CDK1 was upregulated in renal clear cell carcinoma and associated with the prognosis, which was consistent with the results of NCAPG. And we analyzed the correlation between NCAPG and CDK1 by immunohistochemical staining among the 72 cases with renal clear cell carcinoma; the results showed that NCAPG is related with CDK1. Previous studies also showed that CDK1 was upregulated in several cancers and related with the prognosis, such as Piao et al. who found that CDK1 and BUB1 were upregulated and predicted poor prognosis of pancreatic ductal adenocarcinoma [27] and Zou et al. who also identified that CDK1, CCNB1, and CCNB2 were the prognostic biomarkers and correlated with immune infiltration in hepatocellular carcinoma [28]. Moreover, Izadi et al. identified that CDK1 might be a potential target for treatment of breast cancer [29]. All the above results implied that NCAPG and CDK1 were upregulated in various cancers and related the prognosis, including renal clear cell carcinoma. And knockdown NCAPG could significantly restrain the proliferation and progression, NCAPG was tightly related with CDK1, and NCAPG/CDK1 complex might be the internal mechanism of NCAPG promoting the proliferation of renal clear cell carcinoma.

In summary, we preliminary proposed that NCAPG promoted the proliferation of renal clear cell carcinoma via mediating CDK1; however, the conclusion needs further verification and consolidation. The limitations of this study are obvious. Single center and small sample faded this conclusion; more experiments of mechanism in vivo and in vitro need to be performed to further consolidate our conclusion. The next aim of our study is to explore the specific relationship between NCAPG and CDK1; the effects of NCAPG/CDK1 complex on the invasion and progression of renal clear cell carcinoma will be the next direction.

## Figures and Tables

**Table 1:** Relationships of NCAPG and clinicopathological characteristics in 72 patients with clear cell renal cell carcinoma.

Feature	All (*n* = 72)	NCAPG expression		*χ* ^2^	*P*
		Low	High		
		*n* = 32	*n* = 40		
Age (year)				2.185	0.139
<55	38	20	18		
≥55	34	12	22		
Gender				0.340	0.560
Male	40	19	21		
Female	32	13	19		
Tumor grade				0.044	0.833
Low	37	16	21		
High	35	16	19		
Tumor size				6.675	0.010∗
<4 cm	35	21	14		
≥4 cm	37	11	26		

**Table 2:** Relationships of NCAPG and CDK1 in 72 patients with clear cell renal cell carcinoma.

All (*n* = 72)	NCAPG		*X* ^2^	*P*	Pearson	Spearman
	Low	High				
	32	40				
CDK1			10.286	0.001	0.378	0.378
Low 30	20	10				
High 42	12	30				

## Data Availability

All data and material are available.
